# MetaMQAP: A meta-server for the quality assessment of protein models

**DOI:** 10.1186/1471-2105-9-403

**Published:** 2008-09-29

**Authors:** Marcin Pawlowski, Michal J Gajda, Ryszard Matlak, Janusz M Bujnicki

**Affiliations:** 1 Laboratory of Bioinformatics and Protein Engineering, International Institute of Molecular and Cell Biology, Trojdena 4, PL-02-109 Warsaw, Poland; 2 Laboratory of Bioinformatics, Institute of Molecular Biology and Biotechnology, Faculty of Biology, Adam Mickiewicz University, Umultowska 89, PL-61-614 Poznan, Poland

## Abstract

**Background:**

Computational models of protein structure are usually inaccurate and exhibit significant deviations from the true structure. The utility of models depends on the degree of these deviations. A number of predictive methods have been developed to discriminate between the globally incorrect and approximately correct models. However, only a few methods predict correctness of different parts of computational models. Several Model Quality Assessment Programs (MQAPs) have been developed to detect local inaccuracies in unrefined crystallographic models, but it is not known if they are useful for computational models, which usually exhibit different and much more severe errors.

**Results:**

The ability to identify local errors in models was tested for eight MQAPs: VERIFY3D, PROSA, BALA, ANOLEA, PROVE, TUNE, REFINER, PROQRES on 8251 models from the CASP-5 and CASP-6 experiments, by calculating the Spearman's rank correlation coefficients between per-residue scores of these methods and local deviations between C-alpha atoms in the models vs. experimental structures. As a reference, we calculated the value of correlation between the local deviations and trivial features that can be calculated for each residue directly from the models, i.e. solvent accessibility, depth in the structure, and the number of local and non-local neighbours. We found that absolute correlations of scores returned by the MQAPs and local deviations were poor for all methods. In addition, scores of PROQRES and several other MQAPs strongly correlate with 'trivial' features. Therefore, we developed MetaMQAP, a meta-predictor based on a multivariate regression model, which uses scores of the above-mentioned methods, but in which trivial parameters are controlled. MetaMQAP predicts the absolute deviation (in Ångströms) of individual C-alpha atoms between the model and the unknown true structure as well as global deviations (expressed as root mean square deviation and GDT_TS scores). Local model accuracy predicted by MetaMQAP shows an impressive correlation coefficient of 0.7 with true deviations from native structures, a significant improvement over all constituent primary MQAP scores. The global MetaMQAP score is correlated with model GDT_TS on the level of 0.89.

**Conclusion:**

Finally, we compared our method with the MQAPs that scored best in the 7th edition of CASP, using CASP7 server models (not included in the MetaMQAP training set) as the test data. In our benchmark, MetaMQAP is outperformed only by PCONS6 and method QA_556 – methods that require comparison of multiple alternative models and score each of them depending on its similarity to other models. MetaMQAP is however the best among methods capable of evaluating just single models.

We implemented the MetaMQAP as a web server available for free use by all academic users at the URL

## Background

Evaluation of model accuracy is an essential step in protein structure prediction. The existing methods for quality assessment of protein models (MQAPs) are usually based either on a physical effective energy which can be obtained from fundamental analysis of particle forces or on an empirical pseudo energy derived from known protein structures (review: [1]. So far, most of the development of MQAPs was focused on the global evaluation of protein structure and most of the existing methods were optimized to discriminate between globally correct and incorrect 'decoy' structures rather than to detect correct and incorrect fragments [2,3]. Even for MQAPs that are capable of generating independent evaluations for each amino acid in the protein structure, it is usually recommended that a score is averaged over a long stretch of residues (e.g. 21 amino acids in the case of VERIFY3D [4]). Systematic assessment experiments, e.g. Critical Assessment of techniques for protein Structure Prediction (CASP) and LiveBench demonstrated that models with a correct fold can be confidently recognized, especially by the fold-recognition meta-servers [5,6]. However, comparative models, especially those based on remotely related templates, often exhibit local inaccuracies that are difficult to identify by a global evaluation, in particular misthreadings of short regions (5–10 residues) corresponding to shifted alignments within individual secondary structure elements [7,8].

In CASP5, we proposed that inaccuracies due to local alignment shifts can be identified and corrected by identification of variable conformations in alternative homology models, comparison of their VERIFY3D scores averaged over only 5 neighbouring residues, and construction of hybrid models comprising the best-scoring fragments [9]. Our method (termed the "FRankenstein's monster approach") turned out to consistently produce very accurate models, especially if regions with initially poor scores were systematically varied to generate additional models for evaluation [10]. However, detailed inspection of cases where we failed to identify the most native-like local conformation based on the VERIFY3D score revealed a considerable variation of scores even among models with similar structural features. Therefore, we decided to carry out a systematic evaluation of the capability of VERIFY3D and several other popular MQAPs, including the recently published method PROQRES [11], to identify the best method for prediction of local accuracy of protein models. However, as the work progressed, we realized that none of the MQAPs we analyzed was sufficiently accurate and robust and that they exhibited very different strengths, and weaknesses. This in turn prompted us to develop a new "meta-predictor" specifically optimized to detect local errors.

## Implementation

### Preparation of protein models for the local quality assessment

#### Training data

We downloaded all models generated within the framework of the Critical Assessment of techniques for protein Structure Prediction (CASP) rounds 5 and 6, for cases classified as 'template-based modeling', i.e. 'comparative modeling' and 'fold recognition' [12,13]. In these cases a large fraction of models have a correct fold and exhibit widely varying degree of global and local similarity to the native structure, with some completely wrong models (of incorrect folds). To create the model database we used only models that covered at least 90% residues of the target sequence and did not exhibit any internal deletions (i.e. missing residues were allowed only at the termini). If the CASP target was a multidomain protein, it was split into individual domains, which were then regarded as separate models. Ultimately, we collected 8251 models for 84 CASP5&6 targets. Then these models where superimposed onto their experimentally solved counterparts using LGA [14], routinely used in CASP assessment. For our datasets, the average root mean square deviation (RMSD) on C-α atoms between the models and the templates is 2.00 Å and the average GDT_TS score is 59.

Many of CASP models are 'non-physical' in the sense that they often exhibit steric clashes, non-standard bond lengths and angles, improper stereochemistry or they lack parts of residues (e.g. residues may be reduced to just C-α atoms). Thus, we 'idealized' our CASP5&6 model dataset to minimize the most severe local errors by simply running MODELLER [15] with default options, using the original model as a template to derive spatial restraints to build a refined full-atom model. We want to emphasize that such procedure can lead to false positives in the case of bad regions of a model and false negatives in the case of excellent refined models.

**Figure 1:**
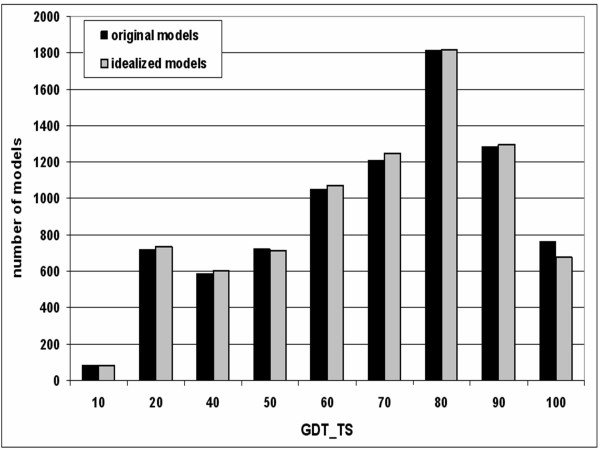
The distribution of GDT_TS scores indicating (dis)similarity between the native structures of CASP targets in the CM and FR(H) categories and the corresponding models: original ones (the CASP5&6 all) and their idealized versions (the CASP5&6+ all).

The average RMS deviation between the idealized models and their original counterparts is 0.33 Å reflecting a slight positional adjustment of the most distorted residues during the idealization. Nonetheless, the GDT_TS score of the idealized models remains 59, the same as for the original models, and the average RMSD with respect to the native structures changes negligibly from 2.00 to 2.01 Å, indicating approximately the same amount of movement towards and away from the native structures during 'idealization'. Analysis of the RMSD and GDT_TS values for models of different accuracy reveals that on the average, our 'idealization' has slightly improved the absolute accuracy of original models with GDT_TS score < 90 (i.e. very good models) and slightly decreased the quality of models with GDT_TS ≥ 90 (Figure 1). Hereafter, the resulting set of models will be referred to as CASP5&6+.

The aim of our analysis was to develop a method that would be able to accurately estimate the deviation of C-α atoms with respect to the corresponding residues in the native structures without any knowledge of the native structure. Despite we introduced 'idealization', we intended to make predictions for the original models. Thus, we trained MetaMQAPII with the deviations between original models (not the idealized models) to the native structures, even though the other component of training was the MQAP score for the idealized models.

#### Test data

In the last part of this article we compare MetaMQAP with CASP7 winners in the MQAP category. Thus, from the CASP7 website we downloaded both the server models and Quality Assessment predictions done by winners of the MQAP category. The accuracy of server models calculated by the LGA method was taken from the CASP7 website [16]. CASP7 server models have been processed in the same way as the training CASP5&6 data, e.g. they have been 'idealized' with MODELLER and scored with MetaMQAP. As with the CASP5&6+ dataset, deviations between the models and the experimental structures were calculated for models before 'idealization'.

### Statistical analyses

We applied wide range of statistical tools: such as Pearson and Spearman's rang correlation, ROC curve analysis, t-test, multivariable regression, and cluster analysis. All statistical analyses were done using STATISTICA 7 software (StatSoft, Inc. Tulsa, OK, USA).

### Model Quality Assessment Programs (MQAPs)

For the evaluation of protein models from the training dataset and for the development of the MetaMQAP we used 8 primary MQAP methods: VERIFY3D [4], PROSA2003 [17], PROVE [18], ANOLEA [19], BALA-SNAPP [20], TUNE [21], REFINER [22], and PROQRES [11]. VERIFY 3D evaluates the environment of each residue in a model with respect to the expected environment as found in the high resolution X-ray structures. It operates on a '3D-1D profile' of a protein structure, which includes the statistical preferences for the following criteria: the area of the residue that is buried, the fraction of side-chain area that is covered by polar atoms (oxygen and nitrogen), and the local secondary structure [4,23]. In our own experience, VERIFY3D is rather permissive (i.e. detects only relatively major errors, usually related to unusual contacts resulting from misalignments, e.g. burial of charged groups in a hydrophobic core. On the other hand, VERIFY3D often fails to detect errors such as non-physical bond lengths or angles or some steric clashes (e.g. threading of a distorted aliphatic side chain through a distorted aromatic ring could be regarded as 'protein-like' by this method). PROSA 2003 relies on empirical energy potentials derived from pairwise interactions observed in high-resolution protein structures [17]. In our own experience, PROSA 2003 is very strict compared to VERIFY3D, i.e. it often detects even very minor errors, such as distorted geometry of hydrogen-bonded residues, and therefore may be more useful for the evaluation of nearly-native homology models than for the fold-recognition models that are plagued by local errors. ANOLEA is also based on a distance-dependent empirical potential. It evaluates the non-local environment (NLE) of each heavy atom in the model. The NLE is defined as the set of all heavy atoms within the distance of 7 Å that belong to amino acids farther than 11 residues in the analyzed polypeptide. Owing to the focus on non-local contacts, ANOLEA is able to identify some errors that remain undetected both by VERIFY3D and PROSA [19]. PROVE analyzes the packing in protein models by evaluating the regularity of the atom volume, defined by the atom's radius and the planes separating it from other atoms [18]. BALA-SNAPP evaluates the structure by means of a four-body statistical potential, applied to tetrahedral quadruplets or spatially neighbouring residues [24]. TUNE uses a neural network to predict local quality of residue from both a local and non local contact of residues in the model [21]. REFINER is based on a statistical potential, which includes terms such as: contacts potential, long distance potential, hydrogen bonds and burial pseudo energy [22]. Finally, PROQRES is the only method in this set, which has been developed specifically to predict local errors in crude protein models. This method applies a neural network to estimate local structure from: atom-atom contacts, residue-residue contacts, secondary structure context, and solvent accessibility [11].

In the final comparison, we analyzed the results of "blind" assessment done for the CASP7 dataset by 6 methods: QA556 – LEE (unpublished), QA704 – QA-ModFOLD [25,26], a method based on the nFOLD protocol [27], QA633 – PROQ, QA692 – ProQlocal [11], QA634 – PCONS6 a new variant of PCONS [28], QA713 – CircleQA (for more information see CASP7 abstracts website [29])

## Results and discussion

### Preparation of a set of models for evaluation

The evaluation of the capability of MQAPs to predict the local accuracy of protein models requires a carefully prepared dataset. We aimed to identify the most native-like segments in a set of high quality models, in particular those generated by comparative modeling and fold-recognition methods. Therefore, rather than analyzing popular sets of decoys with a clear majority of globally incorrect versions of various protein structures [2,30], we decided to use models of all CASP5&6 targets in the CM and FR(H) categories (corresponding to the 'template-based modeling' category in CASP7). We used only models that covered at least 90% residues of the experimentally solved structure and exhibited no missing internal residues (i.e. deletions were allowed only for the termini) (see Methods). Models from the CM and FR(H) categories are usually based on templates with the same fold as the native structure, and the major reasons of their deviation from the native structure are alignment shifts (misthreading) and/or structural divergence between the target and the used template. These models are 'relatively good' only with respect to the correct position of the backbone atoms in the protein core, but they often contain various errors, such as steric clashes between the side chains, missing side chains, unmodeled residues corresponding to insertions or terminal extensions, and discontinuities in the place of deletions. Such models may be considered native-like in terms of C-α atoms, but non-physical in details. Unfortunately, most MQAP methods were optimized for the structures of crystallographic quality, and all 'non-physical' details contribute to their scores in unpredictable ways – either as very serious errors (e.g. steric clashes in ANOLEA) or as artificially positive elements (e.g. some clashes in VERIFY3D). In addition, CASP models are generated by different modeling protocols which exhibit various peculiarities with respect to inclusion or omission of atoms. Variants include C-α atoms, backbone and C-β atoms, all heavy atoms, or all atoms including hydrogens, or different combinations of the above (i.e. in one model some residues may be complete and others may lack different types of atoms). Obviously, it is very difficult to compare the accuracy of residues modeled at such a different level of precision, even if the aim is to assess the accuracy of C-α coordinates only. Moreover, most MQAPs require complete models, without chain breaks or missing atoms, but often also without any hydrogen atoms. Our CASP7 results (see below) clearly demonstrate that utilization of 'crude' CASP models leads to decreased performance of MQAPs, compared to the 'idealized' variants of the same models.

Taking into account the above-mentioned considerations, we constructed an 'idealized' set of models (hereafter referred to as the CASP5&6+ dataset) using MODELLER [15], which minimizes the violation of stereo-chemical constraints as well as restraints derived from the template and yields the canonical set of atoms for each residue. The restraints were derived from the original CASP models, instead of templates. This procedure reconstructed a heavy-atom representation for all residues except the omitted terminal residues and optimized the bond lengths, angles and packing. On the other hand, the backbone structure of such 'idealized' models maintained the conformation nearly identical to the starting structures (see Methods). We envisage only one situation that can lead to a false significant improvement in an idealized model: If the original model contains big gaps (>>3.6Å) between C-α atoms of adjacent residues (obviously an error, as this should not occur in real proteins), MODELLER will attempt to seal the gap, causing conformational change in the neighboring regions and bringing it closer to what may be a native-like conformation (if the flanking regions have correct conformation). However, such cases are quite rare in practice.

The idealized models were used only to compute MQAP scores, while the deviation between the modeled and experimentally observed positions of C-α atoms (used as a measure of the local quality of the model) was calculated for the original, unmodified models. Additional file 1 shows the distribution of residue deviation in our set of 'original' models.

### Critical assessment of MQAPs

All models in the CASP5&6+ dataset were evaluated with 8 popular MQAP methods that we found to be available for download and local installation: VERIFY3D, PROSA2003, PROVE, ANOLEA, BALA-SNAPP, TUNE, REFINER, PROQRES (see Methods). VERIFY3D, ANOLEA and REFINER report series of "raw" scores for individual residues. PROSA reports the composite score and its two components. ANOLEA and BALA report an additional score corresponding to the number of contacts/neighbours of each residue. TUNE and PROQRES report only a single score for each residue. We also analyzed the correlation between residue deviation with local residue features such as: solvent accessibility calculated using NACCESS [31], residue depth calculated using MSMS [32] as well as with the agreement between secondary structure predicted with PSI-PRED [33] and calculated from the model using DSSP [34]. In addition, we studied the accuracy of a trivial score (calculated directly from the model), based on residue depth in the structure size of the protein and type of an amino acid. TrivialScore divides the residues into 2000 classes based on the 10 bins of model size (number of residues in the model), 10 bins of residue depth in the structure (ResDepth) and 20 bins defined by each amino acid. The predicted TrivialScore values directly correspond to the average residue deviation of residues grouped in a given class. TrivialScore should be regarded as a baseline MQAP, which predicts that on the average residues in the protein core are modeled well, and residues on the surface are modeled poorly.

**Figure 2:**
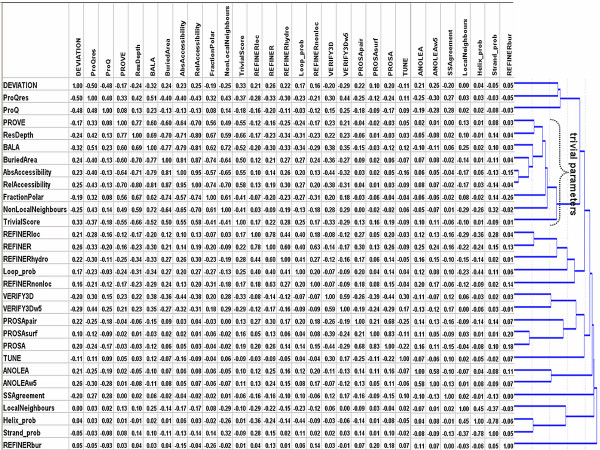
**Absolute value of correlation coefficient of MQAP scores and local residue features with the deviation of residues in the models (all CASP5&6+ all) compared with the native structures. **The dendrogram on the right hand side presents the results of cluster analysis. The linkage between parameters corresponds to the value of (1 – |Spearman's rank correlation coefficient|). On this figure we compare only the primary MQAPs, therefore we do not show results for MetaMQAP that was developed based on these results. The detailed study of MetaMQAP's performance is presented on Figure 3.

Figure 2 illustrates the comparison of the absolute value of Spearman's (R) correlation coefficient between the absolute deviations of the modeled residues from their counterparts in the native structures and all the above-mentioned parameters (MQAPs, local residue features, TrivialScore). In addition, the figure presents the result of a cluster analysis, which shows the relationship between the parameters discussed in this work. We applied the UPMGA method with the value of (1 – |Spearman's rank correlation coefficient|) as the linkage distance. Noteworthy, the Spearman's rank correlation was used here because the relationship between parameters studied here has a non-linear but monotonic character. In such case of nonlinear relationship as a alternative to Spearman's rank correlation the ROC curve analysis can be used. However the ROC curve analysis misses the information required for the cluster analysis presented here.

According to our benchmark (Figure 2), the scores reported by PROQRES exhibit best correlation with the real residue deviation (R = -0.50). This result was expected, since PROQRES is the only method in our test set that has been developed specifically to predict local quality of theoretical models, such as those from CASP. However, our analysis also revealed that PROQRES score performs only slightly better than the global model accuracy (PROQ score – which is identical for all residues in a given model). Apart from PROQ and PROQRES, the correlations of primary MQAP scores with the local accuracy of the model appear very poor – only a few scores exhibit a correlation coefficient above 0.25. The scores that correlate best with the local model accuracy, are BALA, VERIFY3D score averaged in a window of 5 residues (VERIFY3Dw5), ANOLEAw5, and REFINER. It is noteworthy that the 'smoothened' VERIFY3Dw5 score is a much better predictor of local model accuracy than the corresponding "raw" score (VERIFY3D). The same is observed for the ANOLEA and its 'smoothened' variant ANOLEAw5. Another interesting observation is that the composite score reported by PROSA (PROSA) comprises a relatively well-performing component score describing atom-atom interactions (PROSApair) and a much poorer component score describing atom-solvent interactions (PROSAsurf). We also observed that scores reported by different methods are poorly correlated with each other, which provides a stimulus to develop a method that combines strengths of different methods and eliminates the individual weaknesses.

**Figure 3:**
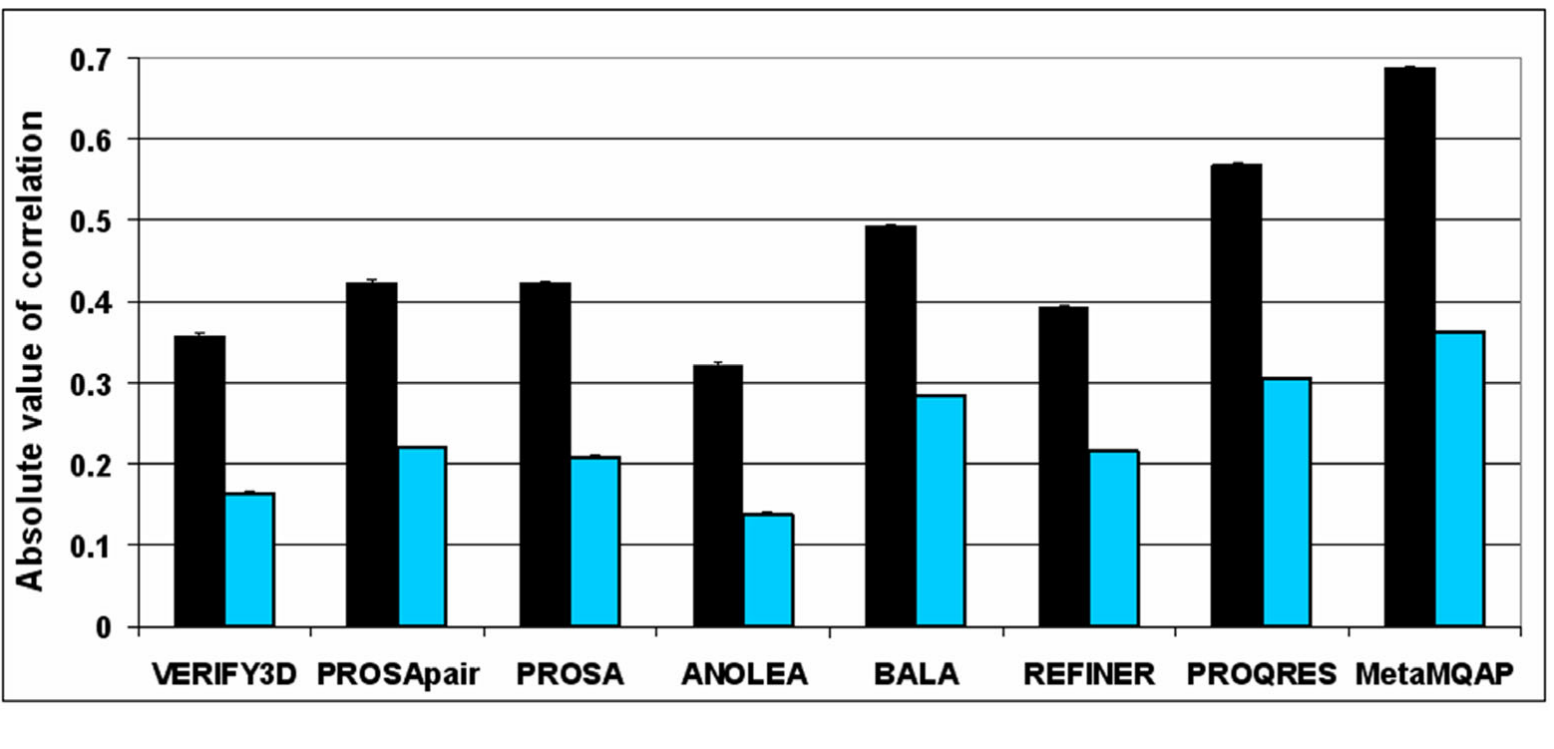
**Benchmark of MQAPs to compare the predictive power of MetaMQAP with other MQAPs (CASP5&6+ test). **Black bars represents absolute values of correlation coefficient of MQAP scores with the deviation of residues in the models compared with the native structures. Blue bars present absolute values of partial correlation, where the following parameters were used as controlling variables: global model quality (GDT_TS) and residue depth in the structure (ResDepth and BuriedArea). At the top of each column, the 95 % confidence interval of correlation is shown.

We were most surprised by a finding that per-residue deviation can be predicted to some extent by using trivial features that can be calculated directly from the model, without any sophisticated MQAPs. Features strongly correlated with per-residue model deviation include NonLocalNeigh, ResDepth, and RelAccessibility, which describe depth or burial of residue in the protein structure. From our results, it appears that one of the best predictors of the local quality of the model is the satisfaction of the residue's preference to be buried in the core (many contacts) or exposed on the surface (few contacts). -In particular, TrivialScore (our baseline 'nonMQAP' score, see METHODS) shows 0.33 correlation with residue deviation (Fig 3), better than most of common MQAPs, e.g. VERIFY3Dw5 or PROSA. Further, we found that PROVE, BALA, and PROQRES show the highest correlation with TrivialScore among all MQAPs tested (correlation coefficient -0,55, -0.52, and -0,37, respectively), which suggests that the scores of these methods mainly indicate the depth of a residue in the structure rather than truly predict the local quality of the model. This phenomenon is even more visible in the results of cluster analysis, which show that MQAPs and residue features group into a few clusters. On of them contains mostly trivial parameters i.e. LocalNeighbours, AbsAccessibility, ResDepth as well as two MQAPs: PROVE and BALA (the second best MQAP according to correlation with residue deviation), suggesting that these MQAPs are biased toward trivial features. Among other clusters, the one grouping PROQRES, PROQ and DEVIATION is most closely related to the cluster of trivial parameters. This means that the residue feature parameters related to depth in the structure are essential for prediction of residue deviation. However, it also means that predictions made by PROQRES may be significantly biased by trivial components.

Summarizing, we observed that the local quality of the model can be predicted to a large extent by consideration of trivial features such as amino acid accessibility and depth in the structure. In other words, as a rule of a thumb, residues buried in the core are on the average predicted more accurately than residues on the surface. Application of sophisticated algorithms implemented in MQAPs provides only a minor added value. To our knowledge this important fact has escaped the attention of developers and users of MQAP. Nonetheless, different MQAP scores are surprisingly poorly correlated with each other, which suggest that these added values might be different in each case. Thus, there is a hope that combination of different MQAPs together with informed analysis of trivial features may provide a much stronger predictor of local model quality than any of these approaches alone.

### Development of a meta predictor

Combination of different sub-optimal predictive methods into 'meta-predictors' has been very successful in structural bioinformatics, in particular in protein and RNA secondary structure prediction [35,36], membrane protein prediction [37], protein fold-recognition [38], and identification of protein domains [39]. Although sometimes even simple averaging shows improvement over the results obtained by the primary methods, there are machine learning techniques that allow more intelligent combination of the best features that different methods have to offer. Here, we used a multivariate linear regression model to develop an MQAP meta-predictor (MetaMQAP). We used the residue deviation as the dependent variable, while MQAPs and residue features were used as predictors.

In order to develop a meta-predictor that would not be biased towards trivial parameters, we applied multivariate linear regression statistical models for selected groups of residues. The selection was based on trivial residue features: global model quality, residue depth in the structure, residue hydrophobicity and secondary structure assignment. In the case of global quality, the residues were first divided into 7 bins, corresponding to 7 groups of models with progressively better PROQ scores (predicted global quality), with bin 1 comprising residues from the 1/7 of models with the worst PROQ scores, bin 7 comprising residues from the 1/7 of models with best PROQ scores etc. Second, residues in each PROQ bin were divided into a new set of 5 bins according to the criterion of ResDepth (thus, yielding 35 bins total). Here, bin 1 comprised 1/5 of most exposed residues (according to ResDepth), while bin 5 comprised 1/5 of most buried residues. Third, we binned the residues according to hydrophobicity (3 bins: hydrophobic, hydrophilic, rest) and fourth – according to secondary structure (3 bins: loop, strand, helix). Ultimately, all residues were divided into 315 groups (7 × 5 × 3 × 3). For each of these groups we created a unique linear regression statistical model to predict residue deviation based on parameters described in Table 1. It is worth mentioning that linear regression assumes that the relationship between dependent variable (residue deviation measured in Å) and independent variables is linear, but this criterion would not be satisfied in our study. Thus, we transformed both deviation and other parameters into ranks from 1 to 100 to amend this problem. Finally, we applied the least squares method to estimate linear regression parameters for each of 315 models. Each of the models was created by STATISTICA 7 software, then the output was parsed by in-house PYTHON scripts.

**Table 1:** Description of scores returned by the primary MQAP methods as well as other local features analyzed in this work.

VERIFY3D	3D-1D profile score for a single residue
VERIFY3Dw5	VERIFY3D score averaged over a 5 residue window
PROSApair	pair energy (atom-atom interactions)
PROSAsurf	surface energy (atom-solvent interactions)
PROSA	combination of PROSApair and PROSAsurf
ANOLEA	distance – dependent empirical potential. It evaluates the non-local environment (NLE) of each heavy atom in the model
ANOLEAw5	ANOLEA score averaged over a 5 residue window
PROVE	average relative volume for all atoms of a residue
BALA	mean of a four-body statistical potential, applied to tetrahedral quadruplets or spatially neighbouring residues
REFINERloc	pseudoenergy of local contacts
REFINERnonloc	pseudoenergy of long-distance contacts
REFINERhydro	pseudoenergy of H-H bond interaction
REFINERbur	pseudoenergy of burial
REFINER	weighted sum of all REFINER pseudoenergies
TUNE	score based on neural network that predict local quality of residue from both a local and non local contact of residues in the mode
FractionPolar	fraction of non-polar residues in area of given residue (ENVIRONMENT)
BuriedArea	burial of the residue (ENVIRONMENT)
LocalNeighbours	number of residues within the distance of 10 Å in space and within 8 residues in the sequence
NonLocalNeighbours	number of residues within the distance of 10 Å in space and more distant than 8 residues in the sequence.
ResDepth	the distance between the C-α atom of a residue and the closest geometrically plausible position of a water molecule on the surface of the protein
PROQ	It is a neural network based predictor that based on a number of structural features predicts the quality of a protein model. ProQ is optimized to find correct models in contrast to other methods which are optimized to find native structures
PROQRES	score based on neural network which estimate local structure from: atom-atom contacts, residue-residue contacts, secondary structure context, and solvent accessibility
AbsAccessibility	absolute value of solvent accessibility for all atom off a residue (according to NACCESS)
RelAccessibility	proportion of absolute solvent accessibility of a given residue to the solvent accessibility of the same type of residue (X) in a model tripeptyde Ala-X-Ala (according to NACCESS)
LoopProb	probability of a loop conformation in secondary structure predicted by PSIPRED
HelixProb	probability of a helical conformation in secondary structure predicted by PSIPRED
StrandProb	probability of an extended conformation in secondary structure predicted by PSIPRED
SSAgreement	agreement between secondary structure predicted by PSIPRED and secondary structure observed in the model (calculated by DSSP)

It must be emphasized that the linear regression statistical models described above can predict rank (1–100) of deviation instead of the deviation in Ångströms, however the rank can be easily transformed into distance. The predicted rank of deviation were assigned to residue deviation according to average residue deviation of residues of the same rang as predicted in training data

In order to train and test the MetaMQAP the dataset of all CASP5&6+ targets was randomly divided into training data (70 targets – 7032 models total) and test data (14 targets – 1219 models total). The training data was used to train the MetaMQAP method (i.e. to calculate the regression models). The test data (called CASP5&6+ test) was used to evaluate MetaMQAP and compare it with the 'primary' MQAPs.

### Accuracy of MetaMQAP

The score reported by MetaMQAP corresponds to the predicted absolute deviation of a C-α atom of each amino acid in the model from its counterpart in the native structure. Some authors average local model accuracy over a 5 or 9 residue window along the protein sequence [9,40]. In this benchmark we decided to average score for each residue over the window of 5 residues. This procedure was applied both to MetaMQAP as well as to other MQAPs. All such smoothed scores show higher correlation coefficient than corresponding raw scores (data not shown), and MetaMQAP shows a considerable improvement in prediction of the local model accuracy in comparison with the best primary MQAPs (Figure 3). The deviation predicted by MetaMQAP correlates better with an observed deviation than a deviation predicted only using primary MQAPs (e.g. 0.57 for the best primary MQAP PROQRES). The difference between MetaMQAP and PROQRES (and other methods) is statistically significant (t-test, p < 0.05) We also computed a partial correlation coefficient between MQAPs and residue deviation. Partial correlation is a method used to describe a relationship between two variables whilst taking away the effects of another variable or variables (called control variables). We selected global model accuracy (GDT_TS of model), residue depth in the structure (ResDepth) and buried area (BuriedArea) as the control variables. As expected, the correlation coefficient of MQAPs with residue deviation decreased significantly, when the effect of control variables has been subtracted. Despite that fact, MetaMQAP has shown the best correlation of 0.36 in comparison to 0.3 observed for PROQRES (t-test, p < 0.05). Table 2 shows the observed residue deviation of residues that were predicted to be of the best and of the worst quality according to MQAPs. The table presents also the true deviation considered here as a reference. The average deviation of 10% top residues (true deviation) is 0.44 Å. The average for 10% top residues according to MetaMQAP is 1.13 Å, which is more similar to the reference average (0.42 Å) than in the case of other MQAPs. Here, MetaMQAP significantly outperforms common MQAPs such as Veryfy3D (avg. deviation 2.36 Å) and ANOLEA (avg. deviation 2.12 Å). The observed differences between MetaMQAP and other MQAPs are statistically significant (t-test, p < 0.05).

**Table 2:** Local (per residue) deviation for the best and worst residues according to different MQAPs.

	10 % highest quality residues		10 % lowest quality residues		
		
Method	average	std. deviation	average	std. deviation	Area under the ROC curve
TRUE DEVIATION	0.44	0.25	31.3	13.24	1.000
VERIFY3D	2.36	4.22	12.23	14.98	0.699
PROSApair	1.83	3.47	11.27	11.86	0.751
PROSA	1.71	2.71	12.98	13.77	0.752
ANOLEA	2.12	3.89	9.17	11.04	0.685
BALA	1.50	2.08	15.19	16.71	0.767
REFINER	1.85	3.71	10.55	11.83	0.732
PROQRES	1.42	2.08	16.45	15.99	0.814
MetaMQAP	1.13	0.82	20.39	16.76	0.875

The table presents average 95 % confidence interval of average and standard deviation for residue deviation. For a well-performing method, the average of 10 % highest quality residues should be low and for the 10 % lowest quality residues it should be high. In general, the bigger the interval between average residue deviation for best and worst quality residues, the more accurate a method (CASP5&6+ test). In addition the area under the ROC curve (3Å cutoff) is shown

The advantage of MetaMQAP is even greater in a context of standard deviation (Table 2). For MetaMQAP, the 10% top-scoring residues exhibit deviation of 1.13 Å +/- std.dev. 0.82, while for PROQRES, its 10% top-scoring residues exhibit deviation of 1.42 Å +- std.dev. 2.08 Å. In comparison, the 10 % truly best residues exhibit deviation of 0.44 Å +- std.dev.0.25.Å, which indicates that MetaMQAP really pushes the prediction close to the limit. These data clearly show, that MetaMQAP is more confident in detecting residues of highest quality than PROQRES or any other method. A similar situation is observed for the residues predicted to be of the lowest quality. The mean deviation of the residues marked as 10% worst by MetaMQAP is 20.39 Å and is significantly higher than mean deviation of 10 % worst residues selected by each of remaining methods, with a deviation of PROQRES (t-test, p < 0.05) at the level of 16.45 Å. It is noteworthy that 10% truly worst residues have avg. deviation of 31.3 Å, which shows a room for further improvement even in the case of our method (avg. deviation 20.39 Å). In addition we also present the ROC curve analysis (cutoff 3Å) which confirms the performance of MetaMQAP and overall ranking of other MQAPs.

**Figure 4:**
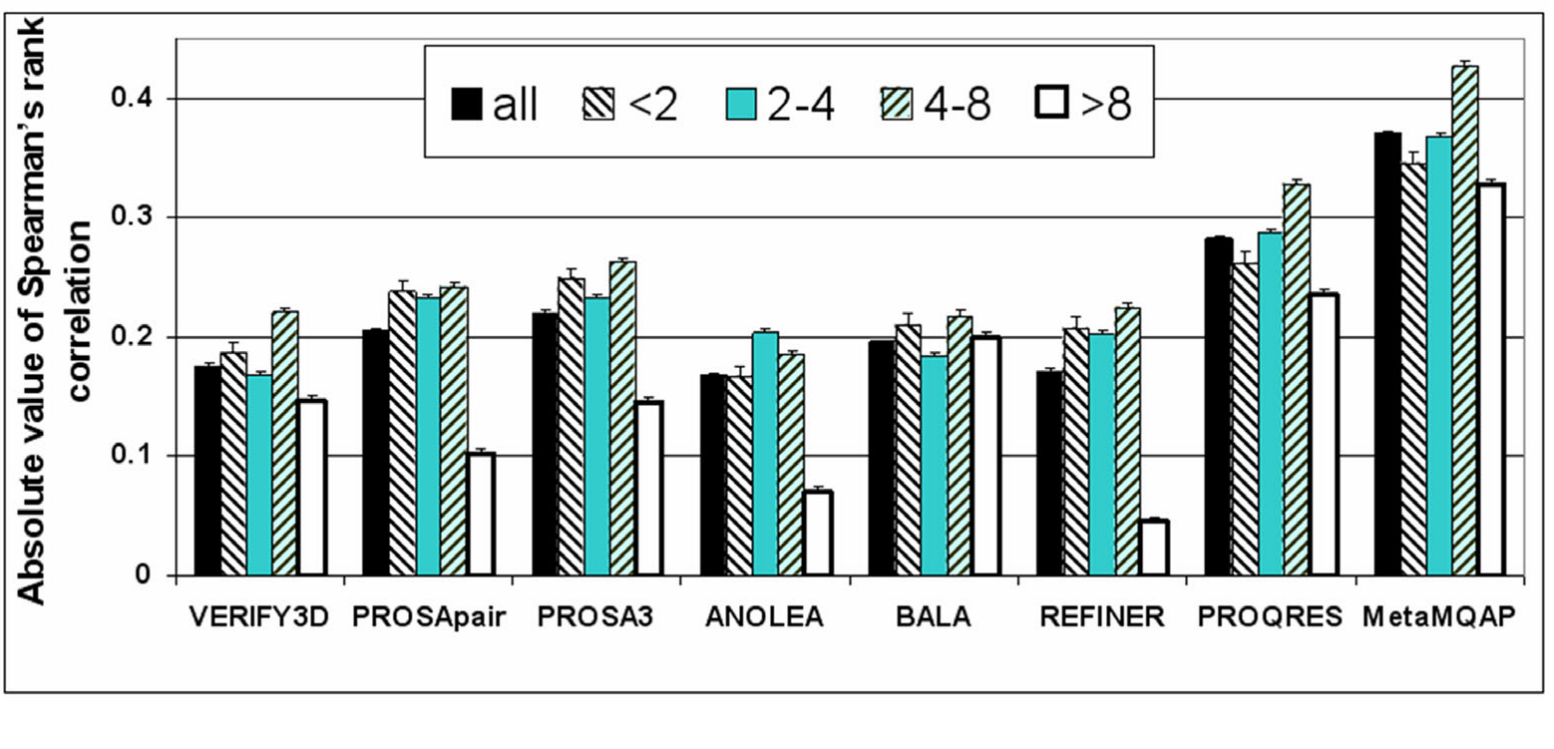
**Absolute value of Spearman's rank correlation between deviation of variants for each residue and their MQAP scores (calculated for the CASP5&6+ test).** The results are showed for all residues as well as classes of residues whose variants in our dataset exhibit mean deviations less than 2 Å, between 2–4 Å, between 4–8 Å and at least 8 Å. At the top of each column the 95 % confidence interval of correlation is shown.

The predicted absolute deviation of individual residues in the model has a similar meaning to the B-factor parameter. It allows for assigning different confidence to regions that are of particular interest for the prediction of biological function of the modeled protein. On the other hand, in some applications it is more important to confidently rank the relative quality of different variants of the same sub-structure in a set of alternative models. The comparison of the ability of MQAPs to estimate the rank of accuracy of the same residue in alternative models is shown in Figure 4.

The study was made for all residues in CASP5&6+ test dataset as well as separately for a few group of residues whose variants in our data set exhibit mean deviations 0–2Å, 2–4Å, 4–8 Å and > 8 Å. Intuitively, it should be easier to rank '4–8Å' sets of residues that include both very accurate and very inaccurate variants than sets '0–2Å' or '>8Å' of residues. In the 0–2 Å range, most variants have similar deviations from the native structure in all models and are typically all very close to the native structure. On the other hand, the set of residues with deviation at least 8 Å usually come from models with globally low quality, so their relative local quality becomes very difficult to predict by all MQAPs. Interestingly, PROSA estimates better rank of variant accuracy than PROSApair in spite of the fact that PROSApair is better correlated with absolute residue deviation than PROSA (see Figure 2). Another useful information for modelers is that PROSA has the same accuracy as PROQRES for the most accurately modeled variants (in the 0–2 Å range). Despite the fact that MetaMQAP was trained to predict absolute residue deviation, here again it outperforms all other MQAPs in ranking different variants of a given residue. The advantage of MetaMQAP over any another method is statistically significant in the case of each studied group of residues (t-test, p < 0.05)

### MetaMQAP in CASP7

A *prototype *of MetaMQAP was one of the methods participating in the Model Quality Assessment contest held within the "7^th ^Critical Assessment of protein Structure Prediction methods" (CASP7). The models submitted by all servers in tertiary structure prediction category were scored by all participating MQAP's, and the assessments were compared with actual model accuracy. Predicted GDT_TS was taken as the global reference score, while single residue deviations from the native structure were taken as the local reference scores [41].

Almost all CASP7 benchmarks have shown that our prototype of MetaMQAP exhibited rather poor accuracy. The phenomenon was observed both for global and local model accuracy. We conclude that it was caused mainly because of two reasons:

First, CASP7 assessors compare MQAP method using Z-score of Pearson's correlation instead of a regular value of the correlation. The Z-score of correlation was calculated in the following way:

$$Z-score=\sqrt{n-3*}(\log(1+r)-\log(1-r)/2)$$

r – correlation coefficient, n – number of scored models

The equations show that the Z-score is proportional to the square root of the number of predictions submitted by an MQAP method. Our method was penalized for providing predictions of only a half of server models, which were full atom models and didn't contain any steric clashes. MetaMQAP (CASP7 group number 038) submitted evaluations for 12259 models while the winning Pcons6 (group 634) scored 23858 server models. Thus, even if the Pearson's correlation between i.e. global model score and GDT_TS would be nearly the same for the two methods, the Z-score is much higher for Pcons6. We conclude that the MQAP scoring system used in CASP7 had an unexpected bias and in our opinion does not necessarily show the discrimination value of the methods tested.

Second, MetaMQAP was trained on CASP5&6+ database containing models idealized by Modeller. In the CASP7 experiment we made a very serious mistake and used MetaMQAP to evaluate models without any idealization. After CASP7 experiment we realized that all models evaluated by our method would be evaluated more accurately, if they were first idealized by Modeller, exactly as we have done for our training set. Such behaviour is now default for  MetaMQAP.

Below we present the benchmark of the *current *version of MetaMQAP on the same set of CASP7 server models that was used in real CASP7 MQAP benchmark. All of these server models were scored by our method after idealization by MODELLER. For both global and local deviations in the benchmark, we use data deposited on CASP7 website (and calculated before idealization) instead of the ones calculated by ourselves.

### Global model accuracy prediction

There are two important questions for which answers are sought. First, how accurate is a given model (in the sense of its GDT_TS score), without considering any alternative models? Second, if there are number of alternative models, how do they compare to each other according to accuracy?

According to the CASP7 Quality Assessment (QA), the four methods that are best in predicting GDT_TS are: QA_556, QA_704, QA_633, and QA_692, while four winners in predicting the rank of alternative models are: QA_556, QA_634, QA_713, and QA_633 [41,42]. To our best knowledge, the QA_556 method is so far unpublished and we could not find any web server, thus it was impossible for us to replicate its results. According to CASP7 abstracts [29], QA_556 is based on global optimalization of a scoring function. A set of models for given target sequence is clustered together and the clusters are evaluated by MODELLER scoring function [43] and/or DFIRE energy [44]. Methods QA_633 (PROQ), QA_634 (PCONS6), and QA_692 (PROQLOCAL) were developed by Elofsson and coworkers [28]. PROQLOCAL combines score of PROQRES and PROQCONF [28].

The PCONS method analyzes the set of protein models and looks for recurring three-dimensional structural patterns [5]. Based on this analysis, each model is assigned a score reflecting how often its three-dimensional structural patterns are present in a whole ensemble of submitted models. It is based on the assumption that recurring patterns are more likely to be correct than patterns than occur only once or in few models. PCONS returns one score reflecting the predicted global quality and scores for all individual residues reflecting their predicted local quality. Unfortunately, the latest version of PCONS -PCONS6 does not allow to submit a single model to score it. It can be used only to score set of models created by the Pcons.net modeling server [45].

The QA_704 method is called QA_ModFold. It combines the scores of MODCHECK, ProQ-LG, ProQ-LX and ModSSEA using a neural network to predict global model accuracy [25]. The method is available as web server [26].

The QA_713 method, also called Circle-QA, predicts model quality in two steps. First, it predicts target difficulty to assign weights for two scoring functions used in a second step. These functions are: 1) agreement between secondary structure predicted for the target sequence and observed in the model, and 2) combined score of burial area, fraction of polar area, and secondary structure. Unfortunately we were not able to find a web server of the method.

Only 3 methods of QA_556, QA_692, and QA_699 belong to both of presented groups of winners (prediction of global model accuracy, AND ranking alternative variants of models for a given target). In a following benchmark we compare MetaMQAP with these 3 methods. We decided to focus our analysis on models that were scored by all the compared MQAPs. Fortunately, almost all of these methods were able to score almost all servers models, so for such a benchmark we used 20486 of 24339 CASP7 servers' models. Additional file 2 presents a number of predictions for each of the considered MQAP methods.

### Prediction of model GDT_TS

**Figure 5:**
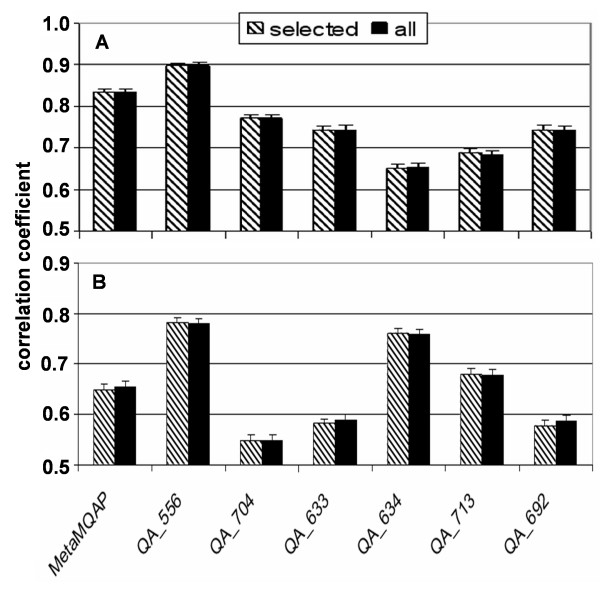
**Correlation between the global score (prediction) and the true model quality (GDT_TS)**. Panel A – Pearson's correlation coefficient between global model accuracy (expressed as a GDT_TS score) and predicted global score (CASP7 server models). Panel B – the mean Spearman's rank between global model accuracy (GDT_TS) and predicted global score of model variants (CASP7 server models). Hatched bars – results for models evaluated by all 7 MQAPs considered here. Black bars results for all CASP7 server models. At the top of each column the 95 % confidence interval of correlation is shown.

Figure 5A shows Pearson's correlation coefficient between GDT_TS and predicted global score for a situation when all predictions are pooled together. We see no significant difference in correlations when we analyse all CASP7 server models or only those scored by all MQAPs. MetaMQAP is 2^nd ^best in predicting global model quality. The score of our method is correlated with GDT_TS on the level of 0.84, while for the most accurate method (QA_556) the correlation coefficient is 0.9

Additional file 3 shows the accuracy of MQAPs as a function of target difficulty. The targets were divided into two different groups: "TBM" (template-based modeling) and "TBM/FM or FM" (template-based modeling or free modeling). We decided to combine TBM/FM and FM targets, because the distribution of GDT_TS score for models submitted for these targets are similar, given that we consider only single domain targets. As expected, all MQAP methods work better for TBM targets. The three most accurate methods in the case are QA_556, MetaMQAP and QA_704. All of these methods exhibit degraded performance for more difficult targets (TBM/FM & FM). Still, our MetaMQAP is also among three best methods, while QA_633 and QA_692 fail in predicting GDT_TS for hardest targets. Surprisingly, for most difficult targets, the size of a model is the best predictor of a model GDT_TS. Actually, it is better than any MQAP score: the correlation with coefficient of -0.6 indicates that the longer a target sequence the poorer are the models. On the other hand, for the TBM group, the size of a target is only weakly correlated with GDT_TS (with a coefficient of 0.1).

Our findings also indicate, that the number of missing residues can greatly influence the MQAP accuracy. Additional file 4 presents the correlation of global MQAP scores with GDT_TS with respect to completeness, measured as the ratio of a number of residues in the model to the total number of residues in the experimentally solved target structure. All methods except QA_556 exhibit significantly decreased accuracy for models which have less than 80% of residues. Therefore we strongly recommend using MetaMQAP only for models, in which no more than 20% of residues are missing.

### Predicted ranking of models

One of the two most important characteristics on an MQAP is the ability to discriminate between better and worse alternative models for a given target sequence. Figure 5B shows the mean Spearman's rank correlation between global model accuracy measured by GDT_TS and the MQAPs' global score. We use Spearman's rank correlation instead of Pearson correlation, because it is more appropriate for ranking alternative models. The Spearman's rank correlation was averaged over all CASP7 targets. We observe that the correlation is similar both when we consider all CASP7 models and when only the models evaluated by all MQAP methods. The correlation of MetaMQAP global score with the rank of model accuracy is 0.65, and it is significantly lower that in the case of method QA_556 (0.78) or QA_634 (0.76). Both of these methods used clustering of large sets of alternative models and then assigned higher scores to models that were members of big clusters. We conclude that availability of numerous alternative models and their clustering based on geometric similarity is a method of choice for ranking of models. This criterion has been implemented earlier for selection of best decoys both from de novo folding [46] and fold-recognition analyses [5].

Additional file 5 presents the ranking abilities of MQAPs relative to the target difficulty. Exactly as before, we grouped single domain CASP7 targets into two classes: 1) TBM and 2) FM and TBM/FM. For easy targets (TBM) the highest accuracy is achieved by QA_556, but the effectiveness of the method significantly decreases for difficult targets (TBM/FM and FM). For the group of hard targets, Pcons6 performs best, because its accuracy is quite insensitive to target difficulty.

**Figure 6:**
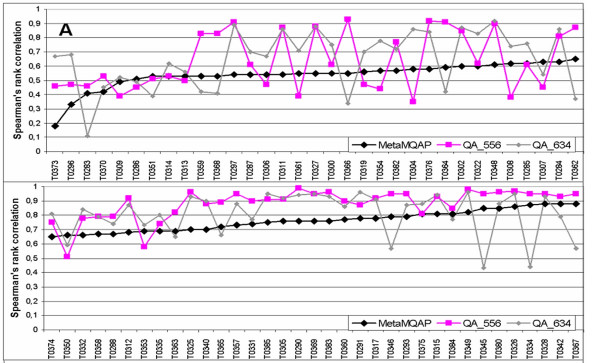
Correlation coefficient between the MetaMQAP global score and the model GDT_TS for each of single domain CASP7 targets.

**Figure 7:**
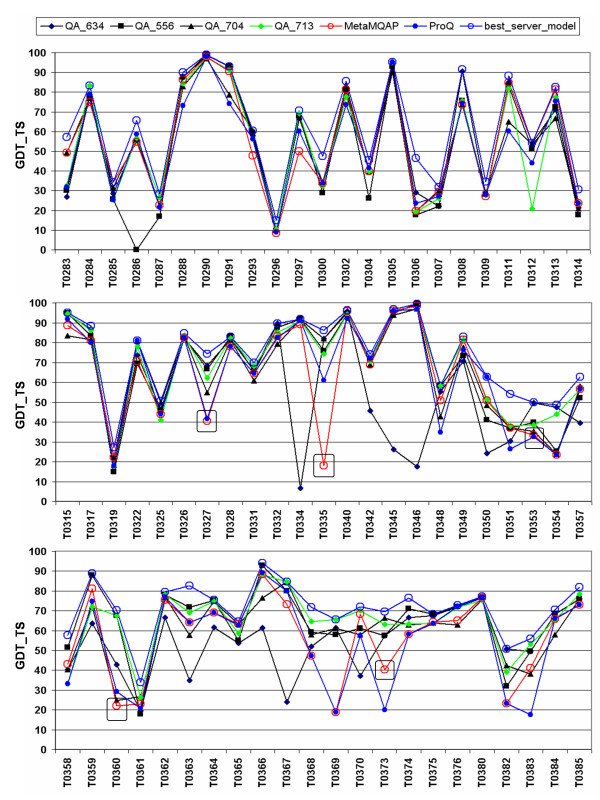
**The ranking abilities of MetaMQAP compared to the best MQAP methods in CASP7 (QA_556 and QA_634, QA_713, QA_704) and ProQ, represented as the GDT_TS score of a model with the highest MQAPs ranking vs. the truly best server model for each target. **Most significant mispredictions made by MetaMQAP are emphasized with red frames. This evaluation was performed on a set of all CASP7 server models.

In Figure 6 and 7 presents an overview of MetaMQAP accuracy over all single domain targets in CASP7. Figure 6 shows the Spearman's rank correlation between the global score of MetaMQAP and the model accuracy. Comparison of accuracy calculated as a difference between the model ranked best by MetaMQAP with the truly most accurate server model is shown in Figure 7. The lowest correlation was observed for target T0373 (with a coefficient of 0.18). The model that was ranked best by MetaMQAP has the GDT_TS score of 40.36, while the best server model has the GDT_TS score of 69.64. Other significant mispredictions of our method are observed for targets T0327, T0353, and T0360, even though the Spearman's rank correlation of these predictions is quite high. In the case of T0373 the highest ranked model contained only 47% of residues, which could have caused MetaMQAP to fail by overpredicting its accuracy. A similar phenomenon is observed for targets T0335 and T0360. MetaMQAP selected model T0335AL333_1, which contains only 39% of residues and T0360TS102_2 which, contains 84% of residues. For the remaining targets T0327 (TBM category) and T0369 (TBM category), ranking errors made by MetaMQAP are difficult to explain. It seems that single outstanding models could have been misevaluated by MetaMQAP assessment.

In Table 3 we present GDT_TS scores of models ranked as best according to MQAPs. We were surprised to see that models ranked as best by PCONS (QA_634 – the best MQAP method in CASP7) have significantly lower overall mean GDT_TS score (57.74) compared to other MQAPs e.g. QA_713 (64.67). This demonstrates that even if an MQAP generates scores that have a good Pearson's correlation coefficient with the true model quality, this method is not necessarily the one most appropriate to select the truly best model. Pearson's correlation coefficient is very sensitive to extreme values, such as those of models with incorrect folds. Contemporary methods can usually predict a correct fold for 'easy' fold-recognition targets, and therefore if alternative models are generated by a number of methods, most of them usually share the same fold and are similar to each other. Thus, a trivial MQAP that only clusters models can reach high correlation coefficient for sets of models for 'easy' targets, only because of its ability to discriminate outliers with clearly non-consensus (and therefore most likely incorrect) folds. In our opinion it would be beneficial to focus the evaluation on the ability of MQAPs to select the most accurate model (or the most accurate parts) in sets of relatively high quality alternative models for a target, and to weigh down the consideration of the ability to discriminate between bad and very bad models (as long as they are discriminated from moderately good and very good models).

**Table 3:** MQAPs ability to detect most accurate model in set of alternative models (analysis performed on a set of all CASP7 server models).

		Percentile of GDT_TS						
		
Method	average GDT_TS of top-ranked models	1	5	25	50 (median)	75	95	99
QA_634	57.74	8.85	18.1	35.38	57.32	73.63	93.70	96.85
QA_556	64.03	13.11	17.66	46.60	68.24	81.01	94.71	99.14
QA_704	62.23	16.65	22.31	40.90	62.97	78.56	93.34	97.10
QA_713	64.67	15.34	21.65	41.78	68.17	81.58	95.15	97.07
MetaMQAP	60.78	14.93	20.39	37.75	64.13	81.11	95.64	98.18
ProQ	59.53	14.78	19.09	32.67	62.56	76.32	94.63	97.28
Best server model	70.93	23.20	30.98	54.63	72.05	85.47	96.13	99.20

The table shows the average GDT_TS score calculated for each top-ranked (according to different MQAPs) model for all targets, as well as cumulative scores for different percentiles of top-ranked models.

### Local accuracy prediction

**Figure 8:**
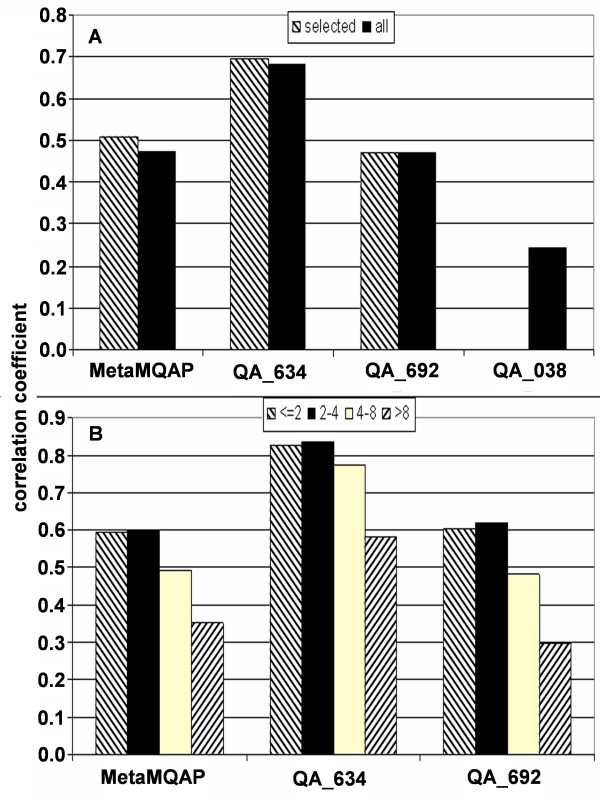
**Correlation between local MQAPs scores and local model quality. Panel A – Pearson's correlation between predicted and observed residue deviation, calculated for CASP7 server models.** Hatched bars – correlation for models evaluated by all 3 MQAPs (MetaMQAP, QA_634, and QA_692). Black bars – correlation for all CASP7 server models. In addition we also present the correlation calculated for our CASP7 predictions – QA_038. In CASP7 experiment, QA_038 submitted scores only for a fraction of models (48% of all scorable residues). **Panel B – **Pearson's correlation between predicted and observed residue deviation as a function of residue difficulty (calculated for CASP7 server models, only for single domain targets). Here we only consider residues scored by all 3 methods (MetaMQAP, QA_634, and QA_692).

In CASP7 experiment only 9 servers competed in predicting the local quality of models. In the following benchmark we compared MetaMQAP with 2 MQAPs that were most accurate in that category: QA_634 (PCONS6) and QA_692 (PROQlocal). Figure 8A presents overall Pearson's correlation between the local residue error and the local MQAP score. Exactly as before, the correlation was calculated for residues in all submitted models (over 7.4 million of residues) or only for the residues in models that were scored by all of the compared methods (6.8 million of residues). We note significant difference between the current version of MetaMQAP and the previous version that did not attempt to 'idealize' models. However, the multi-model comparison method QA_634 has still an advantage, as it uses information unavailable in a single-model regime of MetaMQAP.

Additional file 6 presents the Pearson's correlation between local MQAP scores and true residue deviation as a function of target difficulty. For TBM targets MetaMQAP is the 2nd best method, with the correlation of 0.55. For more difficult targets the accuracy of all methods decreases significantly, but the effect is lowest in the case of PCONS6. Such a result was expected, because for very poor models statistical potentials used by e.g. MetaMQAP or ProQlocal become less accurate and only the information how common a given fragment is in set of alternative models seems to be an effective indicator of model quality.

There is also a clear correlation between MQAP accuracy and residue difficulty (Figure 8B). It can be measured as an average error between instances of a residue in all different models with respect to native structure. The residues were split into four residue difficulty bins: the "easiest" residues are those with a mean deviation in models of less than 2 Å, the "easy but not easiest" have mean deviation between 2–4 Å, and so on. The most difficult residues have mean deviation above 8 Å. For all residue difficulty classes, MetaMQAP shows accuracy comparable to PROQlocal. The accuracy of all methods drops down significantly when the residue mean deviation is > 8 Å.

## Conclusion

We have developed a new method for the quality assessment of protein models, which uses the results of eight other MQAP methods (VERIFY3D, PROSA, BALA, ANOLEA, PROVE, PROQRES, REFINER, and TUNE) and a wide range of local residue features to predict the local deviation of residues in the model from their counterpart in the (unknown) native structure. To our knowledge, this is the first publicly accessible method that attempts to predict the absolute deviation (in Å) for the individual residues in the model in a manner completely independent of used modeling protocol and without any additional alternative models. The development of such methods was recently strongly encouraged in the course of discussions of members of the protein structure prediction community on the FORCASP website [51]. Among similar methods, the ModFold server v1.1 [26] can predict local residue deviations (in the ModFoldClust mode), but only for multiple models.

When our method is compared with the winners of MQAP category in last 7th edition of the CASP experiment [41], our method is outperformed by a few methods that use to their evaluation a large set of alternative models. However, thus far none of these methods has been made available as a public web server. Moreover, these methods require that a potential user provides a large set of models, whose size and diversity would be comparable to all models submitted by all CASP modelers for a given target. It is very unlikely that such an effort would be possible outside the CASP experiment itself. Therefore, MetaMQAP has a potential to become a tool of choice for researchers interested in evaluating just a single model of their target protein, or a few individual models, without necessity to provide a multiplicity of state-of-art models with many different modeling methods.

We hope that our server will be useful for many researchers, perhaps not only computational modelers, but also experimental structural biologists willing to identify confident parts of low-resolution models, e.g. structures generated by automatic tracing of crystallographic electron density maps or preliminary models from an NMR experiment. One possible area of immediate practical application for our method could be the identification of regions of comparative models that deviate less than ~3 Å from the native structure and could be used to solve the crystal structures directly from the diffraction data, using the molecular replacement approach [52-54]. Another possible application is to ensure that the overall quality of the model is sufficient for the intended application and to predicting regions of lower quality for further refinement.

It should be remembered that MetaMQAP is a 'meta-server', and critically depends on the 'primary' methods for the assessment of the local quality of models. The training protocol described in this work can be extended in the future to include additional models (of different quality, similar to QA_634) and additional primary MQAP methods (and structural feature analyses) – both general ones as those that specialize in detection of very minor or very large errors, or deviations of some particular type.

## Availability

**Figure 9:**
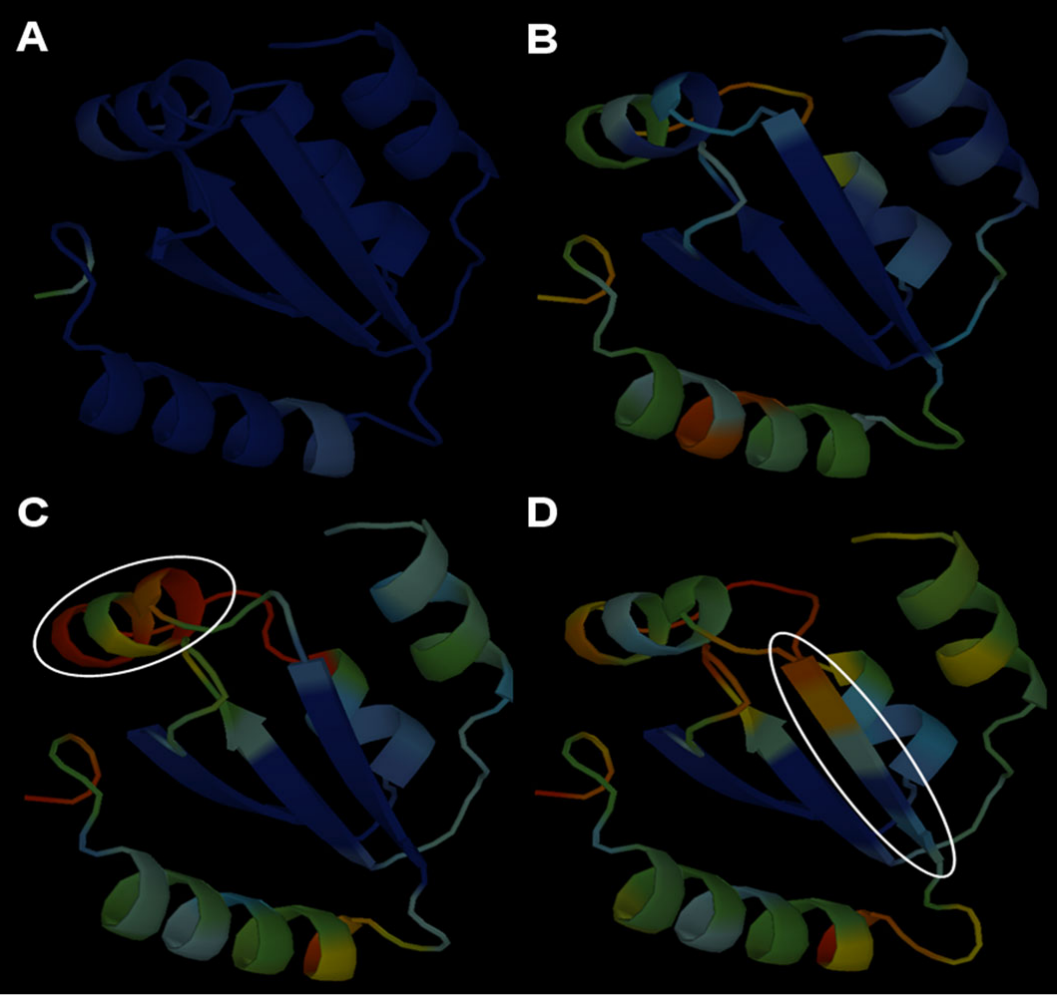
**Visual identification of potential errors in protein models using 'coloring' by MetaMQAP.** The spectrum of colors from blue to red indicates the spectrum of residues predicted to be correct to incorrect. A) The crystal structure of the N-terminal GIY-YIG endonuclease domain of UvrC from *Thermotoga maritima *(PDB code 1ycz). B) A comparative model of the same protein based on an ideal alignment to a closely related structure of UvrC from *Bacillus caldotenax *(PDB code 1yd6). C) & D) Models with local 1 aa alignment shifts indicated by a white ellipse and predicted deviation from the native structure indicated by the shift of the color spectrum from blue towards yellow and red.

We implemented MetaMQAP as a web server available for free use by all academic users at the URL . Any user can submit a model in the Protein Data Bank (PDB) format. After assessment, the web server sends three different files by e-mail. A first file contains a simple report about whether all MQAPs used for computing MetaMQAP score were successfully executed. The second file contains raw scores of primary MQAPs and the deviation predicted by MetaMQAP for each residue, as well as a GDT_TS score predicted for the whole model. The third file contains the model PDB with the temperature (B-factor) fields replaced with the MetaMQAP scores. One option corresponds to linear scaling of values onto the range of 0.00 (predicted no deviation) and 99.99 (predicted deviation ≥ 10 Å), similar to the COLORADO3D server [47]. If an option of 'absolute values' was used, then raw MetaMQAP scores are reported without scaling (i.e. B-factor contains the predicted absolute per-residue deviations in Å). The results can be conveniently visualized e.g. with any macromolecular viewer that allows coloring the structure according to B-factor values (e.g. RasMol [48], PyMol [49], SwissPDBViewer [50] etc.). Per-residue prediction accuracy is visualized as a color in a spectrum between blue (predicted high accuracy), and red (predicted low accuracy). Figure 9 shows an example of three alternative comparative models scored with MetaMQAP, with the results visualized as temperature factors.

Thus far, we have used MetaMQAP in a number of modeling analyses to discriminate between alternative models and to illustrate the uncertainty of different regions in modeled proteins. Table 4 list the published modeling analyses that relied on MetaMQAP that can be used as guides or case studies for potential users.

**Table 4:** Published analyses describing the use of MetaMQAP prior to publication of this article.

**Protein name**	**Protein function**	**Literature reference**
MnmC	Bifunctional tRNA methyltransferase and oxidoreductase	[55]
R.Eco124I	Nuclease/ATPase subunit of Type I restriction-modification system	[56]
Bud23	RNA methyltransferase	[57]
Mom	DNA modification enzyme	[58]
Sgm	RNA methyltransferase	[59]
MiaA, MiaB, MiaE	Enzymes involved in the ms^2^io^6^A biosynthesis pathway: a P-loop NTPase, a Radical SAM enzyme, and a diiron carboxylate oxidase	[60]
M.EcoRII	DNA methyltransferase	[61]
R.MvaI	Restriction endonuclease	[62]
I-Ssp6803I	Homing endonuclease	[63]
R.HphI	Restriction endonuclease	[64]

## Authors' contributions

MP developed MetaMQAP, made statistical analysis and drafted the manuscript. MJG provided advice on development on MetaMQAP and revised the manuscript. RM wrote code to parse MQAPs. JMB conceived of the project, coordinated it, and edited the manuscript. All authors read and approved the final manuscript.

## Supplementary Material

Additional file 1 Absolute deviations (in Å) between the modeled and true positions of C-α atoms of all residues. Deviations were calculated by comparing the CASP5&6 model dataset with the native structures (1110647 residue pairs). Click here for file

Additional file 2 The distribution of prediction of MQAP methods as well as MetaMQAP for CASP7 server methods. Our benchmark database contains 21732 models evaluated by each of the methods. Dataset of models: server CASP7 models Click here for file

Additional file 3 Pearson's correlation between the predicted global accuracy of models (GDT_TS score) and the actual accuracy. Correlation was computed only for models evaluated successfully by all MQAP methods. As a reference, we also present the correlation of a trivial parameter, namely the number of amino acids in the target sequence. Evaluation was performed on a set of all models submitted to CASP7 by servers. Click here for file

Additional file 4 Accuracy of MQAP as a function of model completeness. The picture presents the Pearson's correlation between the global MQAP score and a model global accuracy (GDT_TS score). The correlation was computed only for models scored by all of presented MQAPs. Evaluation on all CASP7 server models. Click here for file

Additional file 5 Mean Spearman's rank correlation between ranking of a model (highest GDT_TS) and a prediction of global model score. TBM, TBM/FM and FM are CASP7 target difficulty classes (TMB = template-based modeling, FM = free modeling). Correlations were calculated only for single domain models evaluated by all MQAP methods. We assumed the 95% confidence interval. Evaluation on a set of all CASP7 server models. Click here for file

Additional file 6 Pearson's correlation between predicted and observed residue deviation. Dashed bars – correlation on a set of single-domain CASP7 models evaluated by all presented MQAPs, black bars – correlation for a set of all single-domain CASP7 server models. Click here for file
