# Supplementary figures and images for: MetaMQAP: A meta-server for the quality assessment of protein models
Source: BMC Bioinformatics. 2008 Sep 29;9:403. doi: 10.1186/1471-2105-9-403 (PMC2573893; doi:10.1186/1471-2105-9-403)

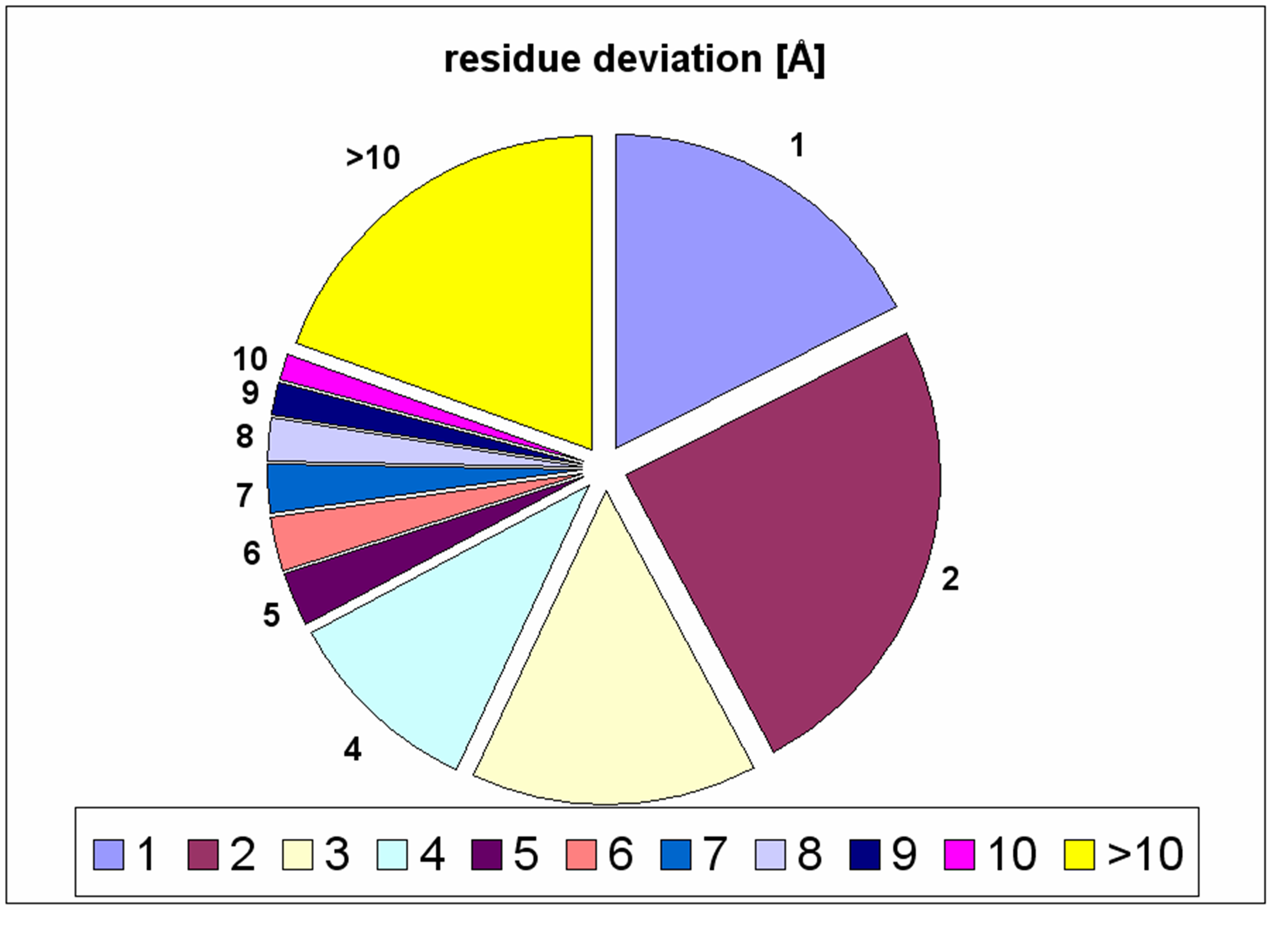

Supplement: Additional file 1 — Absolute deviations (in Å) between the modeled and true positions of C-α atoms of all residues. Deviations were calculated by comparing the CASP5&6 model dataset with the native structures (1110647 residue pairs). [file 1471-2105-9-403-S1.png]

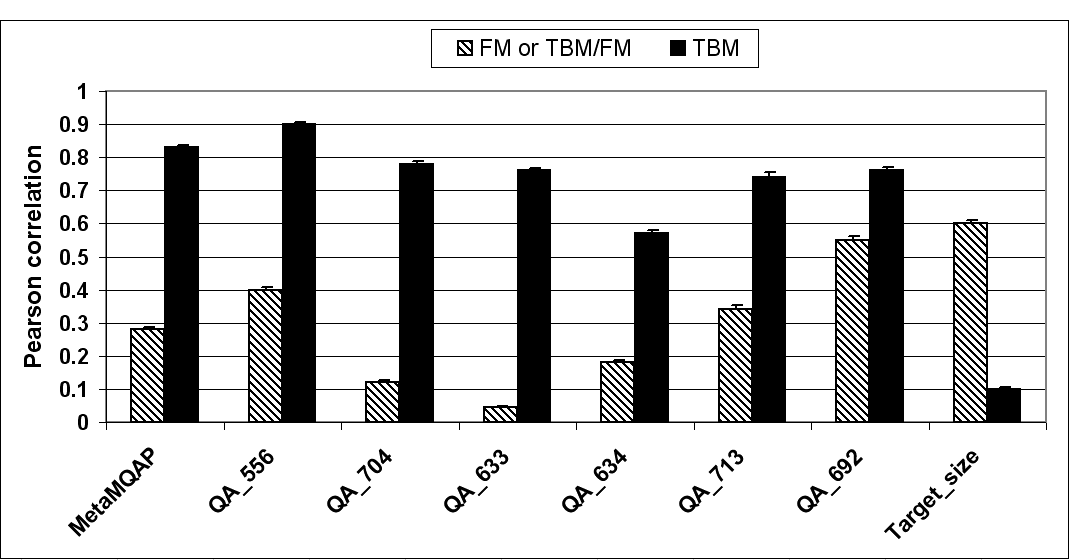

Supplement: Additional file 3 — Pearson's correlation between the predicted global accuracy of models (GDT_TS score) and the actual accuracy. Correlation was computed only for models evaluated successfully by all MQAP methods. As a reference, we also present the correlation of a trivial parameter, namely the number of amino acids in the target sequence. Evaluation was performed on a set of all models submitted to CASP7 by servers. [file 1471-2105-9-403-S3.png]

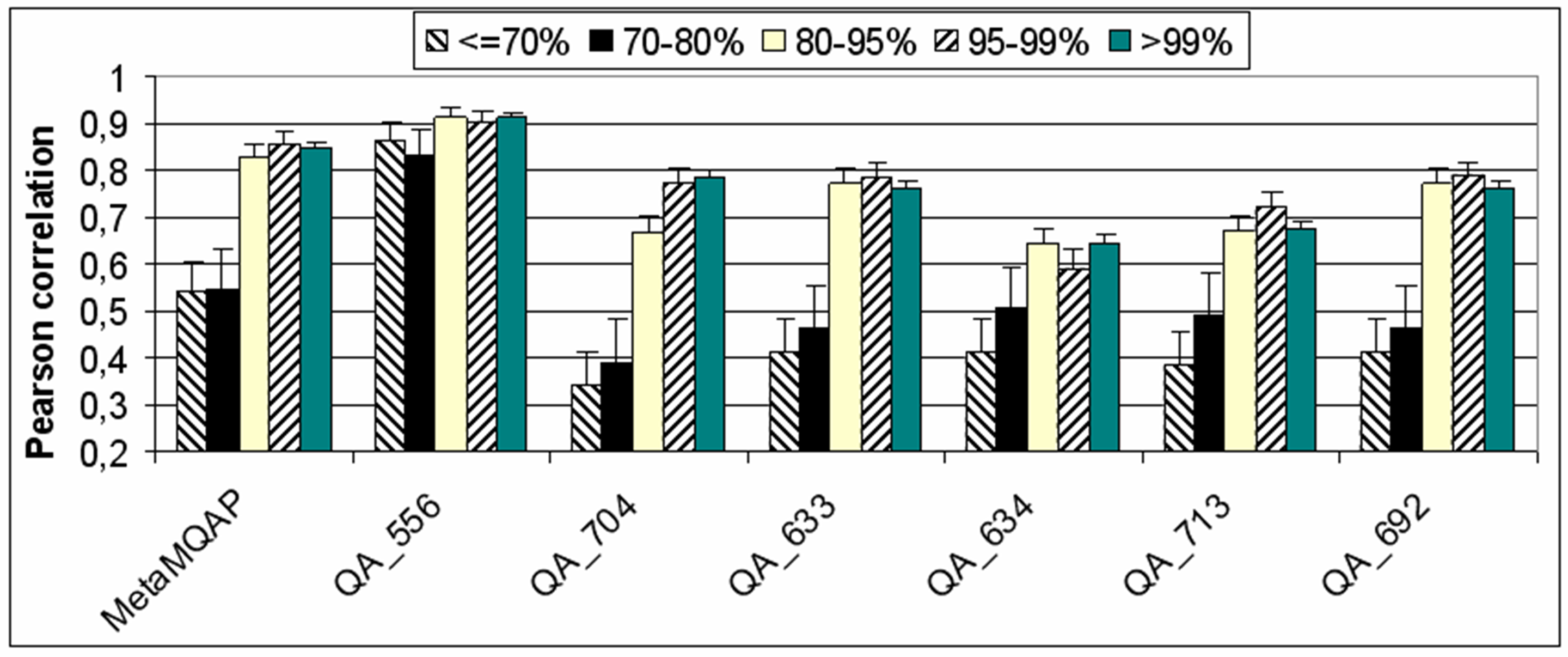

Supplement: Additional file 4 — Accuracy of MQAP as a function of model completeness. The picture presents the Pearson's correlation between the global MQAP score and a model global accuracy (GDT_TS score). The correlation was computed only for models scored by all of presented MQAPs. Evaluation on all CASP7 server models. [file 1471-2105-9-403-S4.png]

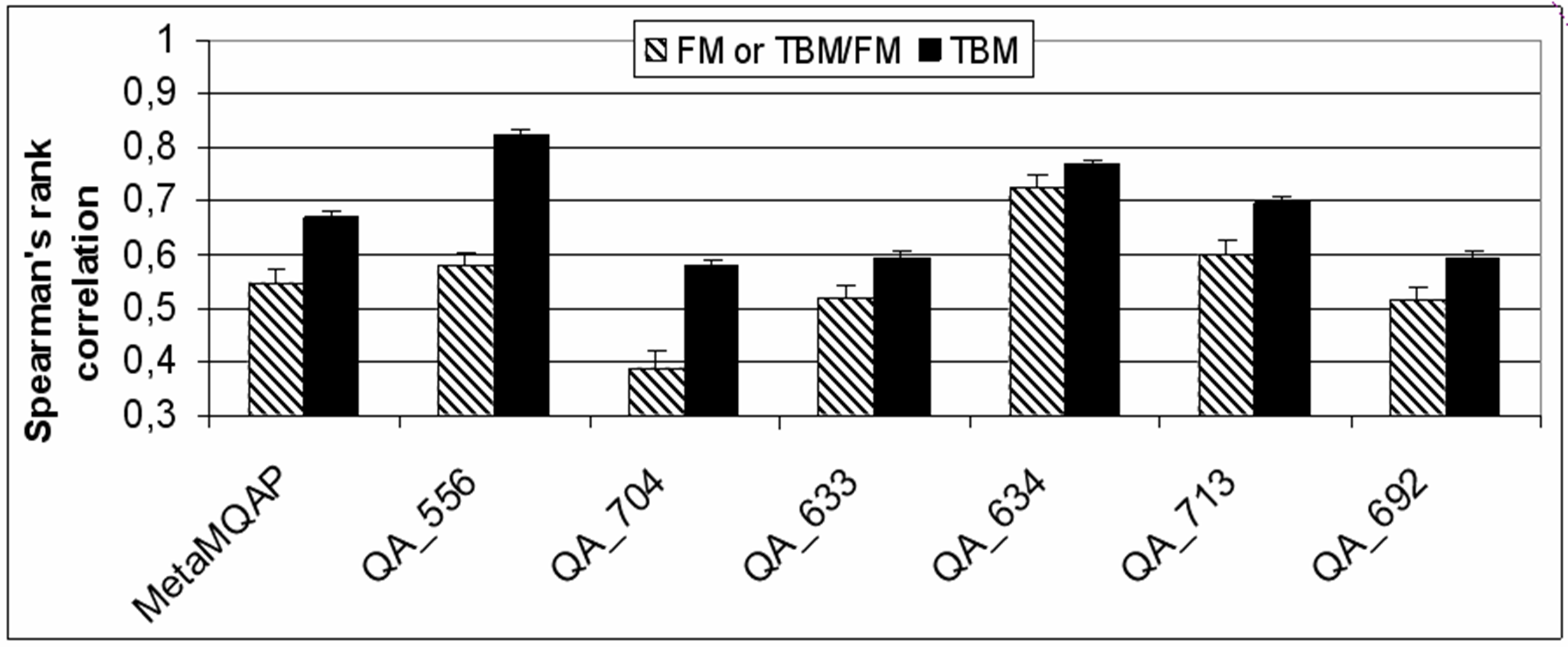

Supplement: Additional file 5 — Mean Spearman's rank correlation between ranking of a model (highest GDT_TS) and a prediction of global model score. TBM, TBM/FM and FM are CASP7 target difficulty classes (TMB = template-based modeling, FM = free modeling). Correlations were calculated only for single domain models evaluated by all MQAP methods. We assumed the 95% confidence interval. Evaluation on a set of all CASP7 server models. [file 1471-2105-9-403-S5.png]

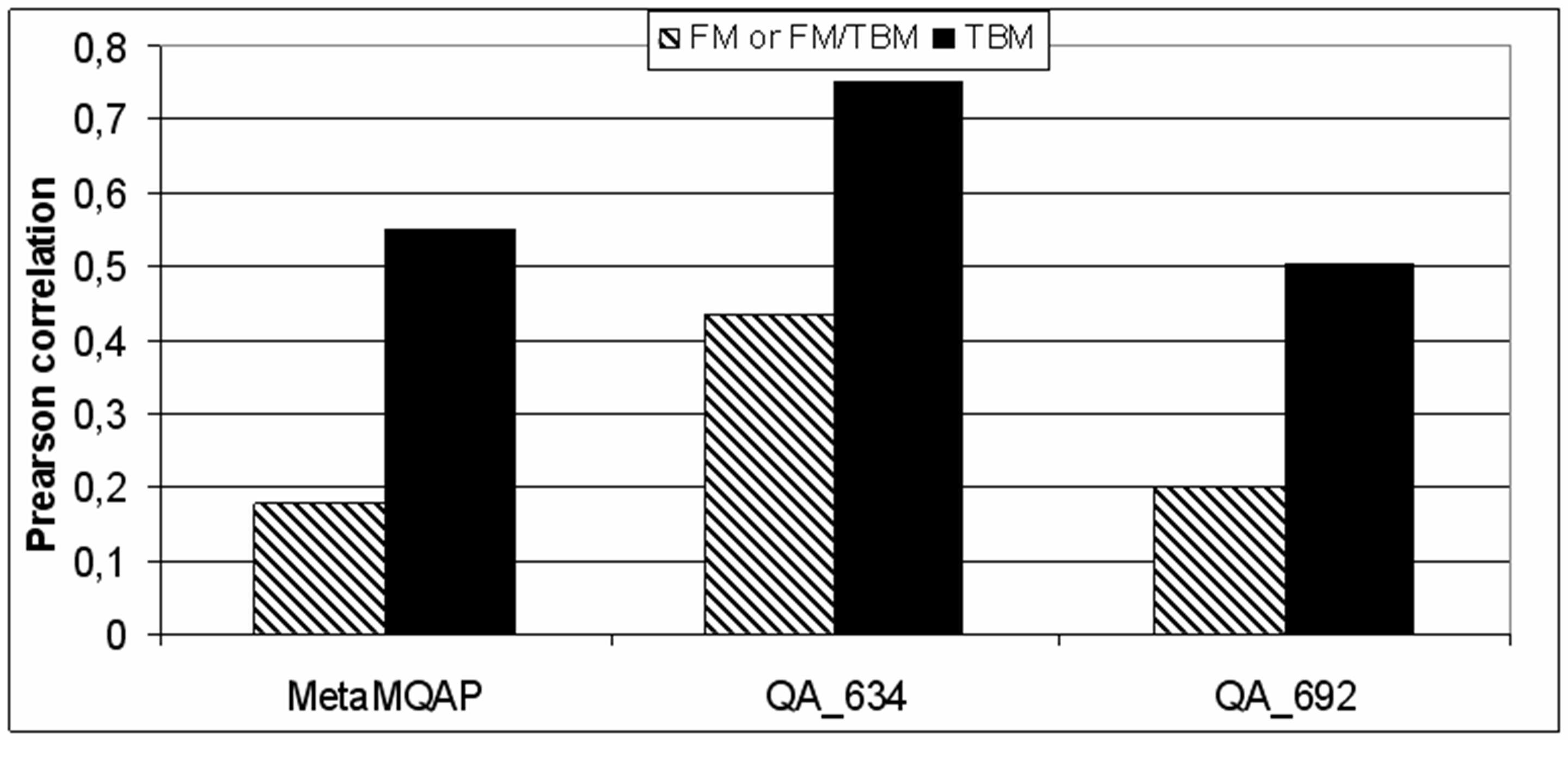

Supplement: Additional file 6 — Pearson's correlation between predicted and observed residue deviation. Dashed bars – correlation on a set of single-domain CASP7 models evaluated by all presented MQAPs, black bars – correlation for a set of all single-domain CASP7 server models. [file 1471-2105-9-403-S6.png]
