# Supplementary material for: MetaMQAP: A meta-server for the quality assessment of protein models
Source: BMC Bioinformatics. 2008 Sep 29;9:403. doi: 10.1186/1471-2105-9-403 (PMC2573893; doi:10.1186/1471-2105-9-403)
Supplement: Additional file 2 — The distribution of prediction of MQAP methods as well as MetaMQAP for CASP7 server methods. Our benchmark database contains 21732 models evaluated by each of the methods. Dataset of models: server CASP7 models [file 1471-2105-9-403-S2.doc]

| **MQAP** | **number of evaluated server models by MQAP** | **Percent of evaluated CASP7 server models** |
| --- | --- | --- |
|  | 24339 | 100,0 |
| MetaMQAP | 24232 | 99,6 |
| QA_556 | 23833 | 97,9 |
| QA_704 | 23832 | 97,9 |
| QA_633 | 23375 | 96,0 |
| QA_692 | 23116 | 95,0 |
| QA_634 | 23858 | 98,0 |
| QA_713 | 23343 | 95,9 |
| **selected** | **21732** | **89,3** |
